# Case-Initiated COVID-19 Contact Tracing Using Anonymous Notifications

**DOI:** 10.2196/20369

**Published:** 2020-06-22

**Authors:** Weibin Cheng, Chun Hao

**Affiliations:** 1 Institute for Healthcare Artificial Intelligence Application Guangdong Second Provincial General Hospital Guangzhou China; 2 Department of Medical Statistics and Sun Yat-sen Global Health Institute School of Public Health & Institute of State Governance Sun Yat-sen University Guangzhou China; 3 Health Information Research Center and Guangdong Key Laboratory of Medicine School of Public Health Sun Yat-sen University Guangzhou China

**Keywords:** COVID-19, surveillance, contact tracing, digital contact tracing, notification, anonymous, labor-saving, stigma, privacy protection

## Abstract

We discuss the concept of a participatory digital contact notification approach to assist tracing of contacts who are exposed to confirmed cases of coronavirus disease (COVID-19); the approach is simple and affordable for countries with limited access to health care resources and advanced technology. The proposed tool serves as a supplemental contract tracing approach to counteract the shortage of health care staff while providing privacy protection for both cases and contacts. This tool can be deployed on the internet or as a plugin for a smartphone app. Confirmed cases with COVID-19 can use this tool to provide contact information (either email addresses or mobile phone numbers) of close contacts. The system will then automatically send a message to the contacts informing them of their contact status, what this status means, the actions that should follow (eg, self-quarantine, respiratory hygiene/cough etiquette), and advice for receiving early care if they develop symptoms. The name of the sender of the notification message by email or mobile phone can be anonymous or not. The message received by the contact contains no disease information but contains a security code for the contact to log on the platform to retrieve the information.

## Introduction

Emerging evidence from the response to coronavirus disease (COVID-19) in China [1], Singapore [2,3], and South Korea [4] has indicated that efficient contact tracing reduces the delay between infection and isolation and accordingly prevents further transmission of the virus [5,6]. Contact tracing, which includes contact identification, listing, and follow-up, is a crucial aspect of epidemic control and is usually driven by health specialists. Contact tracing is a tedious task that requires enormous staff resources, and it is not possible to fully implement contact tracing in regions with widespread transmission. Alternatively, some parts of this task can be substituted and even augmented by technology.

Luca Ferretti and colleagues [1] modelled the potential effect of a digital contact tracing approach that involved training an artificial intelligence algorithm to analyze COVID-19 cases and GPS-based population colocalization information. However, the application of this powerful method may be hindered by advanced technical requirements and violation of privacy regulations. Yasaka and colleagues [7] developed a proof-of-concept smartphone app that allows users to create “checkpoints” for contact tracing and also modelled the effect of such an app under various adoption scenarios. This app respects user privacy by not collecting location information or other personal data. However, this approach relies on high levels of vigilance and willingness to participate among a majority of the population. Another concept of “privacy by design” COVID-19 contact tracing via Bluetooth is being rolled out in Europe [8] which relies on Bluetooth data exchange between two mobile phones to detect whether two people have come into sufficient physical proximity to risk infection, and notifying those who have been in contact with an infected individual who stays anonymous. This type of app has the same constraint of requiring user cooperation to have any chance of success, and it may be more useful in developed countries where smartphones are widely used.

Health care staff resources have been scarce worldwide during the COVID-19 pandemic, especially in developing countries, where technological resources may also be inadequate. We discuss a concept of a contact notification tool to assist tracing of contacts who are exposed to confirmed cases of COVID-19; this tool is simple and affordable for countries with limited access to health care staff and advanced technology.

## Concept of the Tool

The core functionality of our concept is to provide a usable, labor-saving tool for contact tracing by confirmed cases themselves (Table 1). This tool serves as a supplemental contract tracing approach to counteract the shortage of health care staff while providing privacy protection for both cases and contacts. This tool can be deployed on the internet or as a plugin for a smartphone app. Confirmed cases with COVID-19 can use this tool to provide contact information (either email addresses or mobile phone numbers) of close contacts; then, the system will automatically send a message to the contacts informing them of their contact status, what this status means, the actions that should follow (eg, self-quarantine, respiratory hygiene/cough etiquette), and advice for receiving early care if they develop symptoms. The name of the sender of the notification message by email or mobile phone can be anonymous or not. The message received by the contact contains no disease information but contains a security code for the contact to log on the platform to retrieve the information. This approach can prevent reading of the message by people other than the intended recipient. The personal identification data of both the confirmed case and their close contacts will not be recorded during the process. Information provided by confirmed cases will also be encrypted.

**Table 1:** Concept framework for the development of a tool for COVID-19 contact tracing.

Concept	Descriptions and considerations
Products	Smartphone app
	Web-based platform
Operating systems	Windows and Apple
	App compatible with the latest smartphone operating systems
Target users	Suspected and confirmed cases with COVID-19
	People who receive messages from the platform
	People who are concerned about COVID-19 infection
	People who need guidance regarding the control and prevention of COVID-19
	Policy makers and public health specialists
Functionality	Contact notification (eg, email, SMS text messages, autochatbot); can be anonymous or not
	Shared exposure query database
	COVID-19 information hub (optional but strongly recommended)
Application scenarios	Cases when in-person investigation cannot be implemented
	Regions and countries where manual contact tracing is not in place
	Supplement to manual contact tracing, especially in regions of widespread transmission where people with no or mild symptoms are self-quarantined at home
Navigation and use of the tool	Clear definition of close contacts of COVID-19
	Guide for generation of close contacts
	Official email account or telephone number
	Template notification messages
	Rights and responsibilities
	Online consultation
Other considerations	Paramount protection of personal privacy
	Strict adherence to general data protection regulations, careful oversight of data, and effective protections around the use of data
	Guaranteed equity of access to testing and treatment
	Careful design and monitoring to prevent malicious use

Another key functionality is a shared exposure query database for people whose close contacts cannot be reached by messaging. This design is a crowdsourced database that allows patients to input their trajectory information, including public transportation used and times and locations of the patient’s movement prior to and after onset of symptoms. Trajectory information can either be provided by the confirmed patient or obtained during confirmation and uploaded by an epidemiologist. This trajectory information can be visualized on a map, which may be useful for the public to determine their possible exposure to COVID-19. The tool should be simple and easy to access so that it can be used in areas with limited resources. For example, people can visit the website and search by date and route for possible close contact with a patient with COVID-19. This tool can also serve as a hub to access instructions and information about COVID-19 and health services.

## Benefits of the Tool

We envisage that this tool will be useful for people who are concerned about personal privacy and stigmatization related to COVID-19. Persons of Asian descent have faced stigma and discrimination in many places [9]. A person can also be subjected to stigma after they have been released from COVID-19 quarantine even though they are not considered to be at risk of spreading the virus to others. Several digital contact tracing approaches have been explored worldwide [10]. Despite the potential of digital contact tracing, its potential impact is limited because it may conflict with patient data privacy regulations. Our approach is different from other digital contact tracing methods in two ways. One is that it can be function as a website that does not need to be installed on the user’s smartphone. This will substantially lower the threshold for users to access the tool. The other is that no data processing, recording, or analysis occurs on the central server, which reduces concerns regarding personal privacy.

Meanwhile, this approach can be used as a supplement contact tracing tool in regions of widespread transmission where many undiagnosed mild cases are self-isolating at home, such as the United States and United Kingdom. This tool can help save health care resources, freeing staff to provide more urgent testing and clinical care for patients with COVID-19. Although mild cases who self-isolate at home may not associated with the spread of COVID-19, promptly informing contacts of possible risks to take proper precaution measures seems to be necessary and reasonable during a disease outbreak.

## Considerations for Successful Use

Several considerations should be taken into account to guarantee the successful use of this tool. First, the main purpose of this tool is to provide a supplementary approach for patients with COVID-19 to inform their friends, colleagues, and neighbors about possible contact while maintaining their privacy. Second, an accessible COVID-19 testing and care network is needed to guarantee equity of access to testing and treatment to meet the surging demand and relieve the anxiety of people who have received notifications and are anxious about possible infection. Third, privacy protection for both the sender and the recipient are equally important. The General Data Protection Regulation must be strictly followed, and careful oversight and effective protection of the use of data must be ensured. Last, we need to be alert to possible malicious use of this tool. People who do not have COVID-19 may use the system to send messages to others for evil purposes.

## Conclusion

The successful application of this tool relies heavily on public social responsibility and credibility, and it remains to be seen if the public would adopt such a tool and what mechanisms are required to prevent misuse. This is a simple tool that does not require complicated computer techniques despite strict user privacy protection design with respect to countries and regions. Additionally, this tool can help avoid coercive surveillance, facilitate the allocation of health resources, and prioritize clinical service for patients with COVID-19. Information obtained from the platform can also increase our understanding of the epidemiology of COVID-19.

## Abbreviations

COVID-19: coronavirus disease
